# The first morphometric and phylogenetic perspective on molecular epidemiology of *Echinococcus granulosus sensu lato* in stray dogs in a hyperendemic Middle East focus, northwestern Iran

**DOI:** 10.1186/s13071-015-1025-9

**Published:** 2015-08-06

**Authors:** Seyyed Ali Shariatzadeh, Adel Spotin, Shirzad Gholami, Esmaeil Fallah, Teimour Hazratian, Mahmoud Mahami-Oskouei, Fattaneh Montazeri, Hamid Reza Moslemzadeh, Abbas Shahbazi

**Affiliations:** Department of Parasitology and Mycology, Faculty of Medicine, Tabriz University of Medical Sciences, Tabriz, Iran; Immunology Research Center, Tabriz University of Medical Sciences, Tabriz, Iran; Molecular and Cell Biology Research Center, Mazandaran University of Medical Sciences, Sari, Iran; Department of Parasitology, Faculty of Medical Sciences, Tarbiat Modarres University, Tehran, Iran; Tabriz Research Centre of Infectious and Tropical Diseases, Tabriz University of Medical Sciences, Tabriz, Iran

**Keywords:** *Echinococcus granulosus sensu lato*, Stray dogs, G1, G3, G6, Morphometric, Molecular-epidemiology characterization

## Abstract

**Background:**

Hydatidosis is considered to be a neglected cyclo-zoonotic disease in Middle East countries particularly northwestern Iran which is caused by metacestode of tapeworm *Echinococcus granulosus sensu lato*. Human hydatidosis is a high public health priority in the area, however there is little known from a morphometric and phylogenetic perspective on molecular epidemiology of adult *Echinococcus* spp. in Iranian stray dogs.

**Methods:**

80 dogs (38 males and 42 females) were collected during June 2013 to April 2014 in northwestern Iran. The isolated parasites from each dog were distinguished by morphometric keys including small, large hook length and blade length. Subsequently, isolates were confirmed by sequencing of mitochondrial cytochrome oxidase subunit 1 gene.

**Results:**

16 (8 males and 8 females) (Prevalence 20 %) out of 80 dogs were infected to genus *Echinococcus*. With regard to demographic factors, the frequency of parasitism in both male, female adults and their age groups showed no difference (*P* > 0.05). The phylogenetic analyses of *cox1* sequences firmly revealed the 13 sheep strains (G1), one buffalo strain (G3), one camel strain (G6) and one mixed infection. The findings of rostellar hook morphology show an intraspecies variation range among G1 isolates. However, hook measurements in *Echinococcus* derived from G1 (sheep strain) were not a significant difference from those G6 and G3 strains. Six unique haplotypes were identified containing a high range of diversity (Haplotype diversity 0.873 vs. Nucleotide diversity 0.02).

**Conclusions:**

First presence of camel strain (G6) in this region seems to indicate that potential intermediate hosts play a secondary role in the maintenance of camel-dog biology. Current findings have heightened our knowledge about determination of *Echinococcus* prevalence, strains of taxonomy and genotypic trait of parasite in Iranian stray dogs which will also help in the development of strategies for monitoring and control of infected stray dogs in the area.

## Background

Uncontrolled population of infected stray dogs to parasitic infections particularly *Echinococcus* species in areas of increasing densities of human population is a common fact in transmission dynamics of cystic echinococcosis (CE)/hydatidosis.

*Echinococcus* spp. as the most important helminthes-associated zoonosis has considerable impact in disability of worldwide population in endemic areas mainly Russia, Australia, New Zealand, North Africa, South America, China, and the Middle East [1–7].

The overall annual cost of hydatidosis was estimated at US$232.3 million in Iran [8]. Stray dogs as principal definitive hosts serve adult parasites in their intestine while herbivores as intermediate hosts harbor larval stage in their internal organs, especially lung and liver [2]. Therefore, in order to develop control, surveillance system, monitoring and preventive strategies of CE, a better understanding of various aspects of adult *E. granulosus* isolates should be considered sympatrically [9–12].

*Echinococcus granulosus sensu lato* (s. *l*.) isolates show an extensive range of intraspecies variation regarding epidemiology, host specificity, morphology and genetics [13, 14].

Currently, four (G1, G2, G3 and G6) out of ten strains (G1–G10) of genus *Echinococcus* have been genotypically reported from different endemic foci of Iran [10, 15–23]. The infection rate of stray dogs with *E. granulosus* shows a high prevalence of 5 % to 49 % in different parts of Iran [24]. Nonetheless, field study problems such as trapping stray dogs, contamination with viral infections such as rabies and high risk of hydatid infection during experiments, mean there is little known about both the morphometric features and molecular-epidemiology characterization of adult *E. granulosus s. l.* in stray dogs of Iran and even around the world [25–29].

However, many investigators have been successful in their research on the metacestode stages using morphology and/or genotyping of mitochondrial genome in the intermediate hosts including sheep, buffalo, cattle, goat, pig and camels [17, 18, 30–43]. It is important to identify the genetic variation patterns of adult worms of *E. granulosus* to provide a knowledge of existing cycles in endemic foci of Iran, where several intermediate hosts are infected with CE [21, 22].

Therefore, the aim of this study was to investigate the morphometric and phylogenetic perspective on molecular epidemiology of *E. granulosus s. l.* isolates in stray dogs, in order to determine the *Echinococcus* prevalence, strains taxonomy and genotypic feature of isolated parasite which will help in the monitoring and control of infected stray dogs in a hyperendemic focus of Iran.

## Methods

### Study area, sampling and preparation

#### Ethical approval

The animals’ collected were either dead or humanely euthanatized in the course of study with permission from appropriate authorities from the Iranian Environmental Health Organization.

**Fig. 1:**
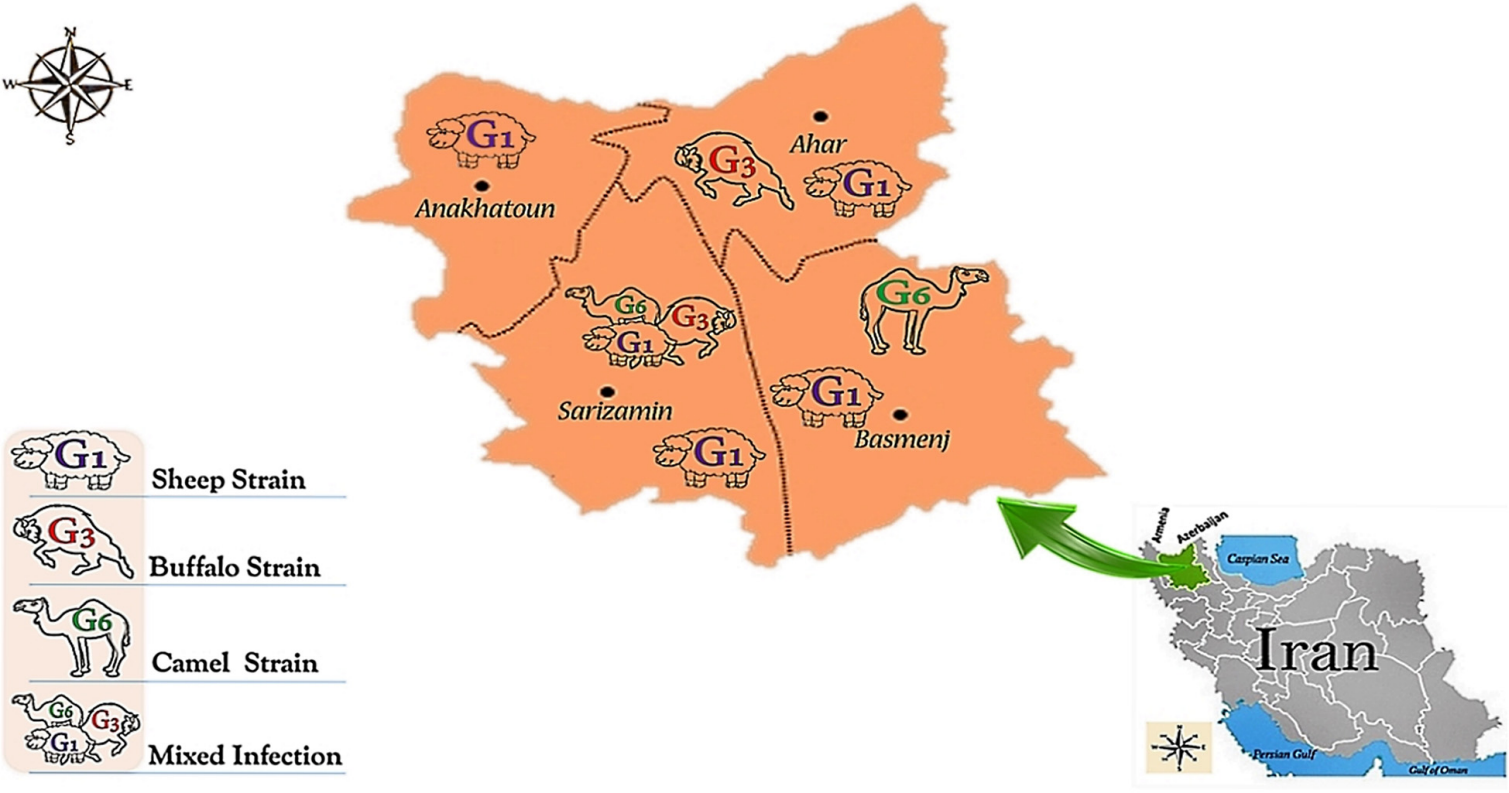
Map of Iran presenting study locations in northwestern Iran

Stray dogs were collected from four different regions of northwestern Iran (Fig. 1): Ahar Basmenj, Anakhatoun and Sarizamin. These are all suburb areas where livestock-farming occurs and the presence of stray and semi-feral dogs was observed. Following necropsy, the intestines of dogs were examined for adult worms of *E. granulosus.*

A total of 80 collected stray dogs (38 males and 42 females) were examined macroscopically during June 2013 to April 2014. First, the age and gender of trapped dogs were determined based on diagnostic criteria [44]. After physical examination, the dog's carcass in the supine position from end sections of ribs longitudinal and perpendicular was slit with the scalpel. Early the mesenteric, and then the beginning of the gastrointestinal tract alimentary canal to the end of anus were removed. To prevent removal of intestinal contents and spread of the infection thread, the double ligature technique was carried out and transferred to the laboratory. Intestines were split in an enamel basin with splitter scissors and its contents were washed with mild stream of water and passed thorough sieves containing 1 mm pores. Isolates were randomly separated from the small intestine of each infected dog and collected in glass containers containing 70 % ethanol.

### Morphometric studies

The isolated worm from each infected dog was identified by diagnostic keys as described by Khalil *et al.* (1994) [45] and Soulsby E.J.L. (1986) [46]. The total length of large (LTL) and small (STL) hooks, blade length of large (LBL) and small (SBL) hooks, the ratio of blade length to total length in large (LBL/LTL) and small (SBL/STL) hooks were measured using a calibrated ocular micrometer at magnifications of 100× (9.5 μm per unit space), 400× (2.5 μm per unit space).

### Total genomic DNA extraction

The measured worms were transferred into a separate tube and washed three times with normal saline and stored in 70 % ethanol until molecular experiments. Genomic DNA was extracted using a High Pure PCR Template Preparation Kit (Roche, Mannheim, Germany) according to the manufacturer’s instructions.

### PCR amplification of mitochondrial genome

The standard PCR was employed to detect *Echinococcus* parasites by targeting *cox1* subunit 1 gene using the primer sets of JB3/JB4.5 [29, 47, 48].

Amplifications were performed under following PCR conditions: 94 °C for 5 min as an initial denaturation, 94 °C for 30 s, 50 °C for 45 s, 72 °C for 35 s in 35 cycles and a final extension at 72 °C for 10 min. PCR products were subjected to electrophoresis in 1.5 % agarose gel and were observed under ultraviolet light after staining for 15 min with (0.5 g/mL) ethidium bromide [47].

### DNA sequencing

PCR products were purified with the Wizard SV Clean-up System (Promega). The final DNA concentration was estimated by comparison with a DNA Ladder Marker (Promega) in 2 % agarosegel. All amplicons were directly sequenced by targeting *cox1* gene in both directions using the mentioned primers by ABIPRISMTM 3130 Genetic Analyzer automated sequencer (Applied Biosystem, USA). Ambiguous (heterozygous) sites were coded using the standard IUPAC codes for combinations of two or more bases. Contigs from all samples were aligned, justified and edited in consensus positions compared to GenBank sequences of all regional species using Sequencher Tmv.4.1.4 Software for PC (Gene Codes Corporation). The diversity testes of analyzed sequences (Haplotype diversity; Hd and Nucleotide diversity: Pi) were determined by DnaSP 5.10.1 software [49].

### Haplotype network and phylogenetic analyses

A network of mitochondrial haplotypes based on the sequences of *cox1* using statistical parsimony was drawn by TCS 1.2 software [50]. The network estimation was run at a 95 % probability limit. Confidence limits with a 95 % confidence interval were established for rates of infection. To evaluate the phylogenetic information provided by *cox1* sequences a Neighbor Net network was built in Splits Tree 4.0 [51] based on genetic distances calculated according to the Kimura-2 parameter model of nucleotide substitutions.

## Results

16 (8 males and 8 females) (Prevalence: 20 %) out of 80 collected stray dogs were infected with genus *Echinococcus.* The number of both infected and non-infected stray dogs based on their age groups and gender are shown in Table 1.

**Table 1:** Age groups and gender frequency of *Echinococcus granulosus* in 80 stray dogs from northwestern Iran

Variables Number of stray dogs	Age groups			Total	Gender		Total
	<3	3-7	>7		Female	Male	
Infected to *E. granulosus*	7 (19.4 %)	7 (22.6 %)	2 (15.4 %)	16	8 (19.0 %)	8 (21.1 %)	64
Non- infected to *E. granulosus*	29 (80.6 %)	24 (77.4 %)	11 (84.6 %)	64	34 (81.0 %)	30 (78.9 %)	16
Total	36	31	13	80	42	38	80

With regard to demographic factors, the frequency of parasitism in male and female adults showed no difference (*P* > 0.05), and in relation to age groups, no meaningful difference was found with contamination rate (*P* > 0.05) (Table 1).

The ranges of LHBL/LHTL and SHBL/SHTL in G1, G3 and G6 strains are summarized in Table 2. The findings of rostellar hook morphology were shown an intraspecies variation range among G1 isolates: LHBL/LHTL = 39.85 ± 3.47 μm to 45.39 ± 3.77 μm and SHBL/SHTL: 29.60 ± 1.98 μm to 39.00 ± 3.50 μm.

**Table 2:** The average morphometric criteria in *Echinococcus granulosus sensu lato* (G1/G3/G6) from infected dogs in the present study

Number of isolated strains from 16 infected dogs	Genotypes	Large hooks [mean ± S.D (range) μm]			Small hooks [mean ± S.D (range) μm]		
		Large hooks total length (L.H.T.L)	Large hooks blade length (L.H.B.L)	LHBL/LHTL %	Short hooks total length (S.H.T.L)	Short hooks blade length (S.H.B.L)	SHBL/SHTL %
1	G1	30.31 ± 2.60 (27.3_34.9)	12.62 ± 1.08 (11.0_14.5)	41.60 ± 1.60	20.98 ± 2.00 (18.0_23.9)	8.10 ± 0.71 (6.8_9.2)	39.00 ± 3.50
2	G1	33.40 ± 3.32 (28.5_38.4)	13.07 ± 0.73 (12.0_14.4)	39.39 ± 3.24	20.93 ± 2.38 (18.4_23.8)	7.53 ± 0.35 (7.1_8.3)	36.45 ± 4.80
3	G1	31.10 ± 2.45 (28.1_36.0)	12.64 ± 1.12 (11.3_14.9)	40.66 ± 2.06	21.60 ± 1.70 (19.0_23.4)	7.96 ± 0.91 (7.0_9.3)	36.90 ± 3.41
4	G1	29.56 ± 1.93 (27.0_33.4)	11.93 ± 0.67 (11.0_12.7)	40.48 ± 2.94	20.96 ± 1.60 (19.4_23.5)	7.13 ± 0.54 (6.1_8.3)	34.14 ± 2.84
5	G1	29.97 ± 3.59 (21.3_35.4)	12.33 ± 0.63 (11.6_23.5)	41.78 ± 6.07	21.67 ± 1.57 (19.4_23.7)	7.89 ± 0.47 (7.12_8.4)	36.68 ± 4.32
6	G1	30.66 ± 0.73 (29.4_31.4)	12.57 ± 0.92 (11.3_13.8)	41.01 ± 2.67	20.78 ± 1.77 (19.0_23.4)	7.21 ± 0.75 (6.2_8.4)	34.70 ± 2.29
7	G1	30.70 ± 2.19 (27.3_34.3)	12.94 ± 1.09 (11.3_14.4)	42.19 ± 2.88	22.37 ± 1.39 (20.3_25.2)	7.00 ± 0.44 (6.3_8.0)	31.34 ± 1.82
8	G1	31.77 ± 1.87 (29.1_34.5)	12.64 ± 1.12 (11_14.7)	39.85 ± 3.47	22.73 ± 1.30 (21.0_25.4)	8.54 ± 0.86 (7.0_9.8)	37.62 ± 3.61
9	G1	30.57 ± 1.35 (27.33_32.0)	12.72 ± 1.12 (10.0_14.0)	41.63 ± 3.65	22.63 ± 1.12 (21.0_24.8)	7.75 ± 0.55 (6.7_8.7)	34.26 ± 1.96
10	G6	32.50 ± 1.35 (30.2_34.4)	13.20 ± 1.26 (11.3_14.8)	40.66 ± 4.06	23.98 ± 1.38 (21.0_24.9)	7.30 ± 0.45 (6.7_8.1)	31.81 ± 1.57
11	G3	30.90 ± 2.26 (27.3_34.6)	13.63 ± 1.07 (11.3_14.9)	44.33 ± 4.58	21.93 ± 2.00 (19.0_34.3)	7.54 ± 1.14 (6.1_9.0)	34.36 ± 3.59
12	G1	32.58 ± 2.94 (29.0_38.0)	13.67 ± 1.08 (11.3_14.9)	40.64 ± 4.28	23.65 ± 1.32 (21.0_25.2)	7.41 ± 0.40 (7.1_8.2)	31.41 ± 1.97
13	G1	31.70 ± 3.10 (26.3_36.0)	13.97 ± 1.04 (12.0_14.6)	44.34 ± 4.60	22.83 ± 1.08 (21.4_24.3)	7.01 ± 0.76 (6.2_8.3)	30.72 ± 2.92
14	G1	30.67 ± 1.80 (27.3_33.4)	13.58 ± 0.76 (12.4_4.8)	44.38 ± 3.05	23.74 ± 1.12 (21.6_25.0)	7.46 ± 0.87 (6.3_9.3)	31.45 ± 3.29
15	G1	28.65 ± 1.11 (27.0_33.1)	12.96 ± 0.76 (12.2_14.7)	45.39 ± 3.77	22.95 ± 1.16 (21.0_25.0)	6.79 ± 0.50 (6.0_7.3)	29.60 ± 1.98
16	G1	31.03 ± 2.46 (29.0_36.2)	13.66 ± 0.75 (12.0_14.3)	44.33 ± 4.79	22.87 ± 1.31 (20.0_24.0)	7.08 ± 0.70 (6.0_8.3)	31.10 ± 3.79

However, hook measurements in *Echinococcus* derived from G1 (sheep strain) were not significantly different from those of G6 and G3 strains.

The morphological findings of rostellar hook obtained from G1 strain in the present and other studies compared to protoscolices derived from different intermediate hosts are shown in Table 3.

**Table 3:** The morphometric characteristics of the G1 genotype derived from dogs in the present study and other studies compared to protoscolices derived from intermediate hosts

Characteristics of hooks in G1 strain	ADULT (Definitive host)					PROTOSCOLECES (Intermediate hosts)		
	Present study (Dog)	Kumaratilake *et al*. (1984) [55] Sheep\dog origin			Hussain *et al*. (2005) [54] Sheep\dog origin	Rajabloo *et al.* (2012) [48] Goat	Thompson *et al*. (1984) ref no, [74] Sheep	Gholami *et al.* (2011) [65] Sheep
		Eastern Australia	Western Australia	Tasmania				
Large hook								
Total length (LTL) μm	31.00 ± 3.21	30.5 ± 1.8	32.6 ± 1.6	34.9 ± 1.8	29.2 ± 1.9	22.93 ± 1.68	25.01 ± 1.1	26.0 ± 1.5
Blade length (LBL) μm	13.04 ± 0.75	12.6 ± 0.9	12.5 ± 0.9	13.8 ± 1.1	---------	11.25 ± 1.35	12.4 ± 1.2	13.4 ± 1.2
LBL/LTL %	42.04 ± 4.79	37.4 ± 3.8	37.1 ± 4.2	39.9 ± 2.2	---------	49.05 ± 4.45	49.4 ± 4.5	51.5 ± 3.4
Small hook								
Total length (STL) μm	22.28 ± 1.31	24.3 ± 2.3	23.1 ± 2.2	30.8 ± 3.1	20.1 ± 2.5	18.7 ± 1.7	21.4 ± 1.5	22.4 ± 1.8
Blade length (SBL) μm	7.48 ± 0.50	9.1 ± 1.0	8.7 ± 1.2	10.4 ± 1.7	---------	8.2 ± 1.23	8.5 ± 09	9.4 ± 1.5
SBL/STL %	33.8 ± 1.98	37.0 ± 3.6	37.9 ± 4.1	33.3 ± 3.5	---------	44.2 ± 6.86	40.6 ± 3.5	41.8 ± 3.8

For all of *Echinococcus* isolates, fragment of 450 bp was successfully amplified within *cox1* gene.

In this survey, *Echinococcus* obtained from each infected dog were directly sequenced and determined firmly as corresponding to the 13 sheep strains (G1) (in Anakhatoun, Ahar, Sarizamin and Basmenj), one buffalo strain (G3) (in Ahar), one camel strain (G6) (in Basmenj) and one mixed infection (in Sarizamin) (Fig. 1). A single-nucleotide variation (transition or transversion mutation) was identified between members of six unique haplotypes. In our targeted regions of *Echinococcus* DNA, insertion or deletion (*Indel*) mutations were not observed in *E. granulosu*s *sensu stricto* (G1, G3) and *E.canadensis* (G6) complexes.

Synonymous substitutions exceeded non-synonymous substitutions in the *cox1* sequences of G1, G3 and G6 genotypes. Within consensus positions, 20 point mutations were observed. Three of these were parsimony-informative sites (24, 34 and 225 bp). Haplotype (gene) diversity (Hd) and Nucleotide diversity (Pi) were 0.873 and 0.02 respectively.

**Fig. 2:**
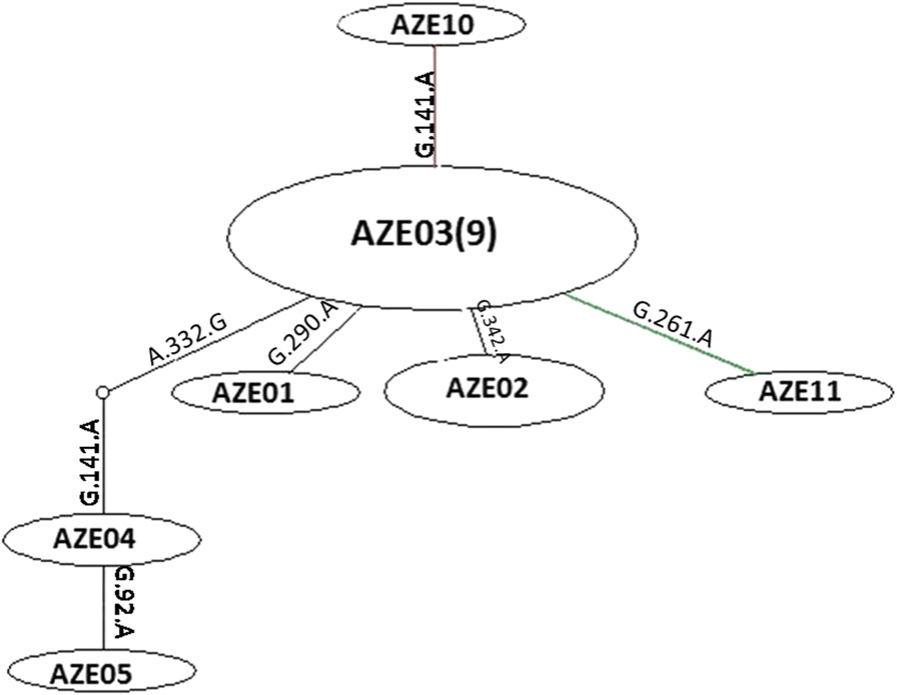
Parsimonious haplotype network of mitochondrial DNA (*Cox1*) obtained from the 16 sequences. The size of the circles approximately indicates the number of individuals, and each mutation event is represented on the lines by a white circle. Haplotype AZE11 in G3 strain and Haplotype AZE10 in G6 strain are charecterized by green and red lines respectively. Haplotypes AZE01, 2, 4 and 5 in G1 strain have linked to common haplotye (AZE03) by black lines

**Fig. 3:**
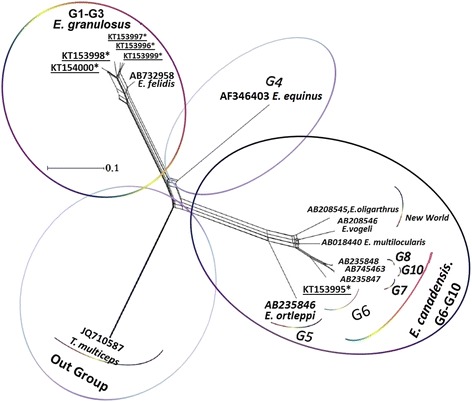
NeighborNet graph according to the Kimura-2 parameter model and sequences of *cox1* gene (mitogenome) of *Echinococcus granulosus sensu lato*. Identified strains in the present study with their submitted sequences are characterized by an asterisk (*) underline

The nine common haplotypes AZE03 (Frequency: 56.25 %) were included without a notable heterogeneity in consensus position (GenBank Accession No; **KP723338**). The six unique haplotypes were included AZE11 (GenBank Accession No; **KT154000**) in G3 (Frequency: 6.26 %), AZE01 (GenBank Accession No; **KT153999**), AZE02 (GenBank Accession No; **KT153998**), AZE04 (GenBank Accession No; **KT153997**), AZE05 (GenBank Accession No; **KT153996**) in G1 (Frequency: 25 %) and AZE10 (GenBank Accession No; **KT153995**) in G6 (Frequency: 6.25 %). To discern a genealogical relationship among the haplotypes, we constructed a statistical parsimony network (Fig. 2). All GenBank accession numbers for the sequences inferred from this study and for the reference genotypes/species used in phylogenetic analysis are shown in Fig. 3.

## Discussion

The prevalence of *Echinococcus* parasites revealed a relatively high level of infection that requires an effective anti-parasite control programme. According to the studies conducted in different countries, the estimated prevalence of dog *Echinococcus* parasites vary from 5 to 70 % [15, 52], and some factors such as geographical location, sampling protocols, demographic factors, anthelmintic usage, and diagnostic techniques are responsible for the wide range of *Echinococcus* prevalence.

The potential role of stray dogs as definitive reservoir hosts for Echinococcosis has been recognized as a significant public health problem worldwide; however few morpho-molecular studies have been carried out based on the identification of different aspects of adult *E. granulosus s. l.* originating from stray dogs [29, 30, 53–55].

In Table 1, the lowest infection (2 of 16) was found in older dogs (>7 years old) than other age groups (<3 and 3-7) because they develop acquired immunity to re-infection in endemic areas although, no meaningful correlation was found between parasitism and age groups based on statistical analysis [56].

The infection rate of *E. granulosus s. l.* among stray dogs was 20 % which there is no concordance with previous study (prevalence 12.5 %) [57]. It is associated with a lack of controlling infected dogs, increasing of unsanitary slaughter around the city and non-normative expulsion of infected viscera of intermediate hosts which are potential ways in transmission of disease [13, 15, 58].

It is worth mentioning that the genotyping of adult *Echinococcus* strains can indicate the scale of parasite biology in the region, while this shows that the intermediate hosts may acquire the infection from neighboring countries/provinces due to their immigrations and importations whereas, the stray dogs are sympatrically limited to an indigenous life [12, 13].

In this study, existence of genotypes G1 and G3 of *E. granulosus* show that sheep and buffalo are unambiguously circulating in the region.

In this study the camel strain was first found in a stray dog. As regards to previous reports, this seems to indicate that the role of secondary intermediate hosts (buffalo/goat/sheep/cattle) which can potentially play a role in the maintenance of camel-dog life cycle [10, 12, 59]. On the one hand, translocation of infected dogs from exceptional regions is the main suspected cause of the introduction of the G6 infection in the region.

In this study, presence of mixed infection of *E. granulosus* has already been explained in the liver and lungs of single animals [60, 61]. This is described by a single infection due to a definitive host concurrently harboring adult worms of the two genotypes or due to consecutive infections of the intermediate host.

To date, the rostellar hooks morphology to be hard, not changeable, quick and inexpensive method is believed as a valid criterion for discriminating *Echinococcus* strains [17, 29, 37, 62–65].

Nevertheless, some researchers believe that employing morphometric criteria alone for the recognition of *E. granulosus* strains are not responsive enough and other complementary characteristics must be considered [66, 67].

The rostellar hook measurements from G1 strain were not considerably different from those G6 and G3 strains whilst, Harandi *et al.* [68] show that the G6 genotype is readily distinguishable from G1 by using both small and large hook lengths in intermediate hosts (hydatid cyst samples of livestock and human origin). They also demonstrated that the total large hook length can help to distinguish the G3 and G6 genotypes. These contradictory results are revealed by two facts. First, the morphometric keys cannot always be considered as a well-known criterion in discrimination of *Echinococcus* strains in both intermediate and/or definitive hosts due to various growth patterns of parasites in developmental stages (metacestode or adult). Second, due to the low number of G3 and G6 strains in this study, it should be investigated on one more sample size.

Generally, the size and shape of hooks are variable through the parasite's development which supports our findings based in Table 3. These differences may explain why dogs are usually infected with collected protoscolices from several hydatid cysts, whilst the sample of protoscoleces for hook measurements frequently comes from a single cyst. However, if contamination of intermediate hosts is achieved through heterogeneous sources [30] it is probable that the hook measurements of adult worms are genetically different from the protoscoleces, and subsequently lead to differences in hook measurements.

High haplotype diversity (Hd 0.873) identified in stray dog population are alerted to pathogenecity range of *E. granulosus*/*E. canadensis* complexes, the creation of emergent strains in under studied areas and also the resistance of adult worms versus host innate immunity responses, including apoptosis [6, 69, 70].

The intraspecies variations among some G1 sequences provide evidence of which mechanisms of slippage, unequal crossing over/transposition and genetic drift/founder effect have led to the variation in *Echinococcus* species [71]. Also, it seems that the lack of any bottleneck effects in the under studied areas and the long term geographic segregation into the regions are probable heterogeneity assumptions [72].

## Conclusions

For the first time, a relatively high prevalence of genus *Echinococcus*, different morphometric of sheep strain (G1) along with various strains (G1/G3/G6 and mixed infection) of *E. granulosus s. l.* were identified and developed by morphometric and molecular-phylogenetic taxonomic aspects in northwestern Iranian stray dogs. These findings are strengthened by our knowledge of educating the public in order to improve hygiene habits, to minimize the parasite’s chance of transmission, to prevent initial contamination of the environment, controlling the size of stray dog populations, and routinely treating dogs with appropriate anthelmintic drugs. Based on recent investigations, further research will be required to determine whether the current EG95 vaccine would be effective against the *E. granulosus s. l.*, or whether it will be necessary, and possible, to develop genotype-specific vaccines [73].
